# Safflower Improves Memory, Learning, and Behavior in Rats Subjected to Sleep Deprivation

**DOI:** 10.7759/cureus.70150

**Published:** 2024-09-25

**Authors:** Muhanned M Alqhtani, Noor A Al Mousa, Noor M Al Zayer, Layan A Al Abbas, Nourah Alamer, Mohammed A Almousa, Yahya M Naguib

**Affiliations:** 1 Internal Medicine Department, King Hamad University Hospital, Manama, BHR; 2 College of Medicine and Health Sciences, Arabian Gulf University, Manama, BHR; 3 Physiology Department, College of Medicine and Health Sciences, Arabian Gulf University, Manama, BHR; 4 Physiology Department, College of Medicine and Medical Sciences, Arabian Gulf University, Manama, BHR; 5 College of Medicine, King Khalid University, Abha, SAU; 6 Internal Medicine, Medical University of Łódź, Łódź, POL; 7 Clinical Physiology Department, Faculty of Medicine, Menoufia University, Shebin El Kom, EGY

**Keywords:** anxiety., stress, learning, memory, safflower, sleep deprivation

## Abstract

Background: Sleep is a physiological process that provides the body with a window for recovery and restoration. Intriguingly, even short-term sleep deprivation can impair brain memory, emotional capacity, information processing, and attention. Safflower (*Carthamus tinctorius L.*) has been shown to attenuate memory loss and improve anxiety and depression.

Objective: This study aims to study the possible therapeutic effect of safflower on sleep deprivation-dependent effects on memory and behavior.

Materials and methods: Thirty young male Wistar albino rats were acclimatized, trained, and then assigned to three random groups: control (C), sleep-deprived (SD), and sleep-deprived Safflower-treated (SD+Sf) groups. Morris Water Maze (MWM) and Elevated Plus Maze (EPM) tests were used to study spatial memory and learning and anxiety-related behavior, respectively, in the study groups.

Results: There was a significant deterioration in learning and memory, as tested by the MWM in the SD group, compared to the C group. This included prolonged test duration, reduced average speed, and longer travel distance. Treatment with safflower significantly improved MWM test performance in the SD+Sf group when compared to the SD group. When compared to the C group, rats in the SD group demonstrated altered EPM test parameters suggestive of anxiety-like behavior. These included spending more time in the closed arms, spending less time in the open arms, and having fewer entries in the open arms. Rats in the SD+Sf group showed improved EPM test parameters when compared to the SD group.

Conclusion: Safflower significantly ameliorated sleep deprivation induced by memory loss and altered behavior. Safflower supplementation may provide potential memory-enhancing and preserving, anxiolytic, and antidepressant therapeutic roles.

## Introduction

Sleep is a physiological process that is fundamental for maintaining various aspects of human health, including proper physical and cognitive functions [1-3]. There are many quantitative and qualitative parameters of the physiological sleep process, any of which can lead to sleep deprivation (SD). Contemporary fast-paced and demanding lifestyles have identified SD as a global concern with complex medical, social, and professional etiologies [4]. The latter particularly applies to sectors such as healthcare, security, and transportation. SD has been linked to deleterious health issues such as memory impairment, learning difficulties, depression, anxiety, increased risk of cardiovascular disorders, metabolic disturbances, immunocompromisation, and all-cause mortality [5]. Moreover, SD has been reported as a cause of low productivity, increased accident rates, and compromised decision-making abilities, thereby impacting both individual and community well-being [6].

Using natural compounds as traditional remedies is a common practice in various communities where this heritage is passed on through generations. Safflower (*Carthamus tinctorius L.*) has been cultivated in many parts of the world and has been used for hundreds of years as a food additive, coloring agent, and medicinal herb [7]. There is an accumulating body of literature addressing the possible therapeutic roles of safflower, including antidepressant-like action, angiogenesis, wound healing, anti-thrombotic, anti-inflammatory, anti-ischemic, and neuroprotective effects [7-12]. However, to the best of our knowledge, there is insufficient data regarding the role of safflower in ameliorating the deleterious consequences of SD on memory and behavior.

Countering the undesirable effects of SD is a priority at both individual and societal levels. Consequently, the aim of the present study was to investigate a possible therapeutic role of treatment with safflower on sleep deprivation-dependent changes in memory and behavior in experimental animals.

## Materials and methods

Ethical considerations

This work was carried out in compliance with the rules and recommendations of the Research and Ethics Committee (REC) at the Arabian Gulf University and in adherence to the Guiding Principles of the Use and Care of Animals published by the National Institute of Health (NIH) [13]. The study received ethical approval from the REC (E22-PI-11-21). All personnel who participated in the laboratory work were certified in laboratory animal handling. At the end of the study, all rats were humanely sacrificed by CO_2_ inhalation [14].

Setting and study animals

The study was conducted in the Faculty of Medicine and Health Sciences, Arabian Gulf University, Kingdom of Bahrain. Thirty male Wistar albino rats (ages ranging from five to six weeks) were used in the present study. Rats were allowed to acclimatize for 10 days. Rats were fed standard laboratory chow ad libitum and were allowed free access to water in an air-conditioned room with a 12-hour light/dark cycle.

Study group

Rats were randomly divided into three experimental groups, each containing 10 rats, as follows: (i) control (C) group - rats were kept in their cages and slept normally, with free access to water and food. (ii) Sleep-deprived (SD) group - rats were subjected to paradoxical SD for 72 hours. Rats also had free access to water and food during the SD period. (iii) Sleep-deprived safflower-treated (SD+Sf) group - rats were subjected to SD for 72 hours and concomitantly received safflower (commercially available) in a daily dose of 600 mg/kg dissolved in distilled water via gastric lavage.

Induction of sleep deprivation

SD was induced according to the validated method of Choi et al. and others [15,16] with slight modification. Rats were kept in a custom-made glass tank (dimensions 120 cm × 40 cm × 40 cm) containing 10 platforms. The platforms were purposefully designed to allow alert standing of each rat without allowing them to sleep. When the rats tended to fall asleep, they lost their balance, fell into the water, and subsequently awoke. The rats could move only by jumping from one platform to another. Before filling the glass tank with water, rats were left in the tank one hour per day during the three days of acclimatization preceding SD. Eventually, the glass tank was filled with water to a level 3 cm below the surface of the platforms.

Assessment of spatial memory and learning

The Morris Water Maze (MWM) was used to study spatial memory and learning [17]. Animals were placed in a pool of opaque-colored water, where they had to swim to a hidden escape platform. The opacity obscured the platform, and the animals would have to rely on scent as an external cue to find the escape route. As the animals became more familiar with the task, they could find the platform more quickly. The following were recorded: the percentage of time spent by animals in the target quadrant (in total), the escape latency (measured in seconds), and the velocity (measured in centimeters per second). Animals were trained for five successive days following the acclimatization period before performing the recorded experiment.

Assessment of anxiety-related behavior

The Elevated Plus Maze (EPM) was used to assess anxiety-related behavior [18]. The EPM had four arms (two open and two closed) arranged in a cross-shape configuration. The ratio of the time spent on the open arms and the time spent on the closed arms was considered a reflection of the animals’ anxiety behavior. Rats were trained for five successive days following the acclimatization period before performing the recorded experiment.

Statistical analysis

Data were analyzed using Origin Pro 2023 software (OriginLab Corporation, MA, US). Following running the normality tests, the analysis of variances (ANOVA) with Tukey’s post-hoc tests was applied. Results were presented as mean ± standard error of the mean (SEM). P-values less than 0.05 were considered significant.

## Results

MWM test results

The recorded means of the total test duration were 18.51±1.98, 56.51±5.21, and 24.90±3.39 sec for the C, SD, and SD+Sf groups, respectively. The mean test duration of the SD group was significantly longer than that of the C group. In comparison, the test duration of the SD+Sf group was significantly shorter when compared to that of the SD group (p-value < 0.05). The average speed of rats in the SD group was significantly slower compared to the average speed of the control rats (1.9±0.2 vs. 7.08±0.25 cm/sec, respectively). Rats in the SD+Sf group reached an average speed of 5.97±0.27 cm/sec, which was significantly faster than rats in the SD group. Rats in the SD group traveled significantly longer distances when compared to their counterparts in the C group (190.38±12.21 centimeters vs. 54.5±6 cm, respectively). Rats in the SD+Sf covered significantly shorter distances (139.61±11.37 cm) when compared to the SD group (Table 1).

**Table 1:** Treatment with safflower enhanced memory and learning in sleep deprived rats. SD: sleep-deprived group, SD+Sf: sleep-deprived safflower-treated group, SEM: standard error of the mean. *Significant when compared to the control group. ^#^Significant when compared to the SD group, p < 0.05, number of rats in each group = 10.

	Control	SD	SD+Sf
Test duration (sec)			
Mean	18.51	56.51*	24.90^#^
SEM	±1.98	±5.21	±3.93
Average speed (cm/sec)			
Mean	7.08	1.9*	5.97^#^
SEM	±0.25	±0.2	±0.27
Total distance travelled (cm)			
Mean	54.5	190.38*	139.61^#^
SEM	±6	±12.21	±11.37

EPM test results

Rats in the SD group spent significantly less time in the open arms of the EPM when compared to the control group (30±6.55 vs. 90±18.36 sec, respectively). Interestingly, rats in the SD+Sf group spent 102.14±35.37 sec in the open arms of the EPM, which was significantly longer when compared to the time spent by the rats in the SD group. On the contrary, the SD group spent significantly longer than the C group in the closed arms of the EPM (270±6.55 vs. 204±20.49 sec, respectively). Rats in the SD+Sf group spent 197.86±35.37 sec in the closed arms of the EPM, which was significantly shorter than the time spent by rats in the SD group. The number of entries in the open arms of the EPM in the SD (6.86±1.24) and the SD+Sf (4.43±0.72) was significantly lower when compared to the corresponding value in the C group (10.3±1.55) (Table 2).

**Table 2:** Safflower counters anxiety and stress in sleep deprived rats. SD: sleep-deprived group, SD+Sf sleep-deprived safflower-treated group, SEM: standard error of mean. *Significant when compared to the control group. ^#^Significant when compared to the SD group, p < 0.05, number of rats in each group = 10.

	Control	SD	SD+Sf
Time spent in open arms (sec)			
Mean	90	30^*^	102.143^#^
SEM	±18.3636	±6.54654	±35.3698
Time spent in closed arms (sec)			
Mean	204	270^*^	197.857^#^
SEM	±20.4912	±6.54654	±35.3698
Number of entries in open arms			
Mean	10.3	6.85714^*^	4.42857^*^
SEM	±1.54955	±1.24267	±0.71903

## Discussion

Sleep is a crucial and cyclic mechanism that occurs in humans and animals and is the most significant restoration and recovery process [19]. To discover any positive impact that natural safflower may have on memory, learning, or behavior in rats subjected to sleep deprivation, both the MWM and the EPM tests were used in this study.

The MWM test aimed to evaluate the effect of safflower on sleep deprivation-induced altered spatial memory and learning. Compared to the SD group, the SD+Sf group performed faster and better in finding the platform, as evident by a significantly shorter test duration (P ≤ 0.05). However, compared to the control (C) group, the SD+Sf group exhibited some spatial learning and memory impairment. Specifically, the SD group roughly took twice as long as the SD+Sf group (56.51 versus 24.90 seconds) to reach the platform. This observed difference may possibly be a consequence of the SD per se [20]. Sleep deprivation has a detrimental effect on memory. Additionally, many studies further stated that long-term memory consolidation is primarily facilitated during sleep [21,22]. Interestingly, the present study showed that although the SD+Sf group traveled a greater distance than the C group, it is still notably less than the SD group, which may reflect the benefit of using safflower to mitigate the effects of SD on overall memory performance in rats. It was previously reported that the aqueous extract of the natural safflower, safflower yellow, alleviated memory impairment in a transgenic mouse model of Alzheimer's disease by depressing the activation of glial cells through its anti-inflammatory effect [23]. Previous studies tried to uncover the mechanism by which sleep deprivation causes memory impairment and anxiety. Neuroinflammation and oxidative stress are believed to be fundamental in the memory impairment and anxiety associated with sleep deprivation [4,24,25]. Hence, ameliorating the expected sleep deprivation-induced oxidative stress and neuroinflammation by using safflower as a natural antioxidant/anti-inflammatory in our study was deemed beneficial [26].

The EPM test was used to assess the anxiety behavior of the study animals, and among the three groups, the SD group spent the longest time in the closed arms, suggesting that the SD rats were significantly more anxious than their control counterparts. On the other hand, the SD+Sf group significantly spent the shortest duration in the closed arms, pointing towards comparatively less anxiety. Spending more time in the open arms relative to the closed arms suggests reduced anxiety [27]. The C, SD, and SD+Sf groups spent a mean of 90, 30 and 102.143 seconds, respectively, in the open arms of the Maze. Compared to the C group, the SD group spent a significantly shorter time in the open arms, reflecting an increased level of anxiety most likely because of SD. Sleep deprivation has been linked generally to distress and specifically to anxiety and depression [28]. The time spent by the SD+Sf group was significantly longer than the SD, which suggests a possible positive effect of safflower in mitigating the effect of SD and as an anxiolytic. Moreover, the SD+Sf group has achieved an even longer mean time when compared with the C group, which adds up/solidifies the support of the anxiolytic effect of safflower. Safflower petal extract was reported to exert anxiolytic and antidepressant effects that were comparable to the traditional anxiolytic and antidepressant drugs diazepam and nortriptyline in rats [29]. The number of entries in the open arms is a complementary measure of anxiety in rodents [27]. The higher number of entries indicates a lower anxiety level, and vice versa. In this study, the C group had the least level of anxiety, as evidenced by the highest number of entries. Surprisingly, in comparison with the C group, both the SD and SD+Sf groups had a significantly lower number of entries, but the SD+Sf group did not demonstrate any significant difference from the SD group. Nevertheless, this could be due to the longer time spent in the open arm by the SD+Sf rats.

Limitations

Despite the observational nature of the study results, they remain interesting. Future work should study the potential mechanisms by which safflower affects the brain. Additionally, the active ingredients of safflower should be purified to maximize their therapeutic benefits.

## Conclusions

The findings of this study add to the body of literature regarding the negative impact of sleep deprivation on memory and behavior. It suggests that safflower is beneficial in counteracting the detrimental effects of sleep deprivation on memory and behavior in rats. Further research is warranted to elucidate the underlying mechanisms and optimize the dosage and administration of safflower active ingredients for maximum effectiveness.
